# Dietary Factors Impact on the Association between *CTSS* Variants and Obesity Related Traits

**DOI:** 10.1371/journal.pone.0040394

**Published:** 2012-07-23

**Authors:** Henri Hooton, Lars Ängquist, Claus Holst, Jorg Hager, Francis Rousseau, Rikke D. Hansen, Anne Tjønneland, Nina Roswall, Daphne L. van der A, Kim Overvad, Marianne Uhre Jakobsen, Heiner Boeing, Karina Meidtner, Domenico Palli, Giovanna Masala, Nabila Bouatia-Naji, Wim H. M. Saris, Edith J. M. Feskens, Nicolas J. Wareham, Karani S. Vimaleswaran, Dominique Langin, Ruth J. F. Loos, Thorkild I. A. Sørensen, Karine Clément

**Affiliations:** 1 Institut national de la santé et de la recherché médicale (INSERM), U872, Nutriomique, Paris, France; Université Pierre et Marie Curie-Paris Paris, France, 6, Centre de Recherche des Cordeliers, U872, Paris, France; Université Paris Descartes, Paris, France; 2 Institute of Preventive Medicine, Copenhagen University Hospital, Copenhagen, Denmark; 3 Centre national de genotypage (CNG), Paris, France; 4 INTEGRAGEN, Paris, France; 5 Danish Cancer Society, Institute of Cancer Epidemiology, Copenhagen, Denmark; 6 National Institute for Public Health and the Environment (RIVM), Bilthoven, The Netherlands; 7 Department of Cardiology, Aalborg Hospital, Aarhus University Hospital, Aalborg, Denmark; 8 Department of Clinical Epidemiology, Aarhus University Hospital, Aalborg, Denmark; 9 Department of Epidemiology, German Institute of Human Nutrition, Potsdam, Germany; 10 Molecular and Nutritional Epidemiology Unit, Cancer Research and Prevention Institute (ISPO), Florence, Italy; 11 Medical Research Council (MRC) Epidemiology Unit, Institute of Metabolic Science, Addenbrooke’s Hospital, Cambridge, United Kingdom; 12 Universite Paris-Descartes, Paris, France; 13 Institut national de la santé et de la recherché médicale (INSERM) U970 Paris Cardiovascular Research Centre, Paris, France; 14 Department of Human Biology, Nutrition and Toxicology Research Institute of Maastricht (NUTRIM), Maastricht, The Netherlands; 15 Division of Human Nutrition, Wageningen University, Wageningen, The Netherlands; 16 Centre for Paediatric Epidemiology and Biostatistics and MRC Centre of Epidemiology for Child Health, UCL Institute of Child Health, London, United Kingdom; 17 Institut national de la santé et de la recherché médicale (INSERM), U1048, Obesity Research Laboratory, Team 4, I2 MC, Institute of Metabolic and Cardiovascular Diseases, Toulouse, France; 18 University of Toulouse, U1048, Paul Sabatier University, Toulouse, France; 19 Clinical Biochemistry Department, Toulouse University Hospitals, Toulouse, France; 20 The Novo Nordisk Foundation Center for Basic Metabolic Research, Universiy of Copenhagen, Copenhagen, Denmark; 21 Assistance Publique-Hôpitaux de Paris, Hôpital Pitié-Salpêtrière, Département de Nutrition, Paris, France; Centre de Recherche en Nutrition Humaine-Ile de France, Paris, France; Sudbury Regional Hospital, Canada

## Abstract

**Background/Aims:**

Cathepsin S, a protein coded by the *CTSS* gene, is implicated in adipose tissue biology–this protein enhances adipose tissue development. Our hypothesis is that common variants in *CTSS* play a role in body weight regulation and in the development of obesity and that these effects are influenced by dietary factors–increased by high protein, glycemic index and energy diets.

**Methods:**

Four tag SNPs (rs7511673, rs11576175, rs10888390 and rs1136774) were selected to capture all common variation in the *CTSS* region. Association between these four SNPs and several adiposity measurements (BMI, waist circumference, waist for given BMI and being a weight gainer–experiencing the greatest degree of unexplained annual weight gain during follow-up or not) given, where applicable, both as baseline values and gain during the study period (6–8 years) were tested in 11,091 European individuals (linear or logistic regression models). We also examined the interaction between the *CTSS* variants and dietary factors–energy density, protein content (in grams or in % of total energy intake) and glycemic index–on these four adiposity phenotypes.

**Results:**

We found several associations between *CTSS* polymorphisms and anthropometric traits including baseline BMI (rs11576175 (SNP N°2), p = 0.02, β = −0.2446), and waist change over time (rs7511673 (SNP N°1), p = 0.01, β = −0.0433 and rs10888390 (SNP N°3), p = 0.04, β = −0.0342). In interaction with the percentage of proteins contained in the diet, rs11576175 (SNP N°2) was also associated with the risk of being a weight gainer (p_interaction_ = 0.01, OR = 1.0526)–the risk of being a weight gainer increased with the percentage of proteins contained in the diet.

**Conclusion:**

*CTSS* variants seem to be nominally associated to obesity related traits and this association may be modified by dietary protein intake.

## Introduction

Obesity is caused by a large number of factors that can be summarized as an interaction between an unhealthy environment and a predisposing genetic background. There is a wide spectrum of obesity-susceptibility ranging from strictly genetically determined obesity to fully environmentally determined obesity with most individuals containing a complex mix of these factors–i.e. many individual effects of genes, environmental influences and the interaction between these two. While epidemiological approaches, including twin studies, have shown that genetic factors may account for as much as 57 to 86% of body mass index (BMI) variations [1], there have been more than 450 genes referenced in the national canter for biotechnology information “NCBI gene” database (http://www.ncbi.nlm.nih.gov/sites/entrez) as being associated with obesity, but each of these genes individually has a small effect on BMI variance. This is very well illustrated by the results of genome wide association studies on large populations which have investigated the implication of several hundreds of thousands of single nucleotide polymorphisms (SNPs) in BMI variance [2]–[4]. New variants influencing BMI have been discovered, yet these variants, even in combination, only explain a very small part of the observed BMI variations and therefore it seems that most of the causal variants remain to be discovered. It is most likely that the largest part of the variance of BMI or other adiposity related traits attributable to genetic factors is due to a large number of variants, each of which has a very small effect [5]–[7]. Nevertheless, it seems that genes influencing BMI and those influencing waist circumference and adiposity may belong to a different pool of genes [8]–[10]. Furthermore, the genes involved in BMI and changes in BMI over time might also belong to a different set of genes [1]. However, the possibility that some genes may have a pleiotropic effect on several adiposity phenotypes should not be excluded.

Understanding the mechanisms that underlie development of adipose tissue will contribute to the identification of novel candidate genes involved in BMI and fat mass variations during life. Using a large scale transcriptomic approach in human adipose tissue, we previously identified Cathepsin S as a putative novel biomarker of adiposity [11] produced by adipose tissue. Expression of the *CTSS* gene, encoding for Cathepsin S, in adipose tissue correlates with BMI in obese and lean subjects. Clinical studies also revealed that Cathepsin S circulating levels were correlated with BMI and triglycerides [11], [12]. Furthermore, both *CTSS* adipose tissue expression and Cathepsin S systemic circulating levels were significantly modulated by weight variations either induced by dietary change or bariatric surgery in independent studies [12], [13]. *In vitro* studies showed that this protease also has a local role on adipose tissue. In particular, Cathepsin S contributes to the stimulation of adipocyte differentiation by degrading fibronectin, one of the main components of extra cellular matrix [14]. *In vitro* studies also showed that *CTSS* expression and Cathepsin S secretion in adipose tissue were induced by LPS, TNF-α, and IL-1β, proinflammatory factors that are secreted by cells such as macrophages or smooth muscle cells [11]. In addition Cathepsin S belongs to a family of cystein protease that includes other proteases involved in the development of obesity. In particular, *CTSK*−/− [15] and *CTSL*−/− [16] mice are protected against diet induced obesity. These animals also have improved glucose metabolism related parameters [17].

While the metabolic phenotype of *CTSS*−/− mice is currently under investigation, it is not known whether *CTSS* variants could influence obesity-related phenotypes.

We recently found an association between obesity related phenotypes and rs2424577 [18], a variant located in *CST3*, the gene that encodes Cystatin C, which is the main endogenous enzymatic inhibitor of Cathepsins [19]–[21].

A genetic study carried out by our team showed an association between several SNPs in *CTSS* and metabolic features in women. Rs11576175 was found to be associated with Apo A1 and HDL levels in a group of lean women from the SUVIMAX [22], [23] study; rs10888390, rs10888394 and rs1136774 were found to be associated with Apo A1 circulating levels in a group of obese women [24]. However no consistent association was found between *CTSS* variants and BMI in this study, irrespective of the relatively large sample size (N = 2368 unrelated lean and obese individuals).

Based on these findings, we hypothesize that genetic variation at the *CTSS* locus might influence obesity related phenotypes and their variation over time. We investigated four distinct phenotypes–BMI (body mass index measured in kilograms per squared meters), body fat distribution (measured by waist circumference and waist circumference for given BMI– based on sex-study stratified initial regressions of waist vs. BMI), change in weight during follow-up (either as a quantitative outcome or as a binary weight gainers indicator–experiencing the greatest degree of unexplained annual weight gain during follow-up or not), and change in body fat distribution during follow-up (measured by change in waist circumference and waist circumference for given BMI during follow-up). These four types of phenotypes were chosen since they might be influenced by different sets of genes, although they are all in some way related to adiposity. We addressed this question in a subset of the EPIC [25] cohorts, within the DiOGenes [26], [27] (Diet Obesity and genes) project where both possible associations corresponding to *CTSS*-SNP main effects and some SNP-dietary interactions (GI, protein intake and energy density) were investigated. These dietary factors were chosen since several studies have suggested that diets high in protein and low in GI were beneficial for obesity prevention and weight control by enhancing satiety leading to a decreased energy intake [28], [29].

## Methods

### Ethics Statement

EPIC study has been approved by local review board of all participating institutions, namely the Florence Local Health Authority Ethical Committee (Italy), the Ethics Committee of the Norwich District Health Authority (UK), the Medical Ethics Committee of TNO (Netherlands Organisation for Applied Scientific Research) (the Netherlands), the Ethics Committee of the Medical Association of the State of Brandenburg (Germany), and the Danish National Committee on Biomedical Research Ethics (Denmark). Written informed consent has been obtained from all participants before joining EPIC study.

### Participants

Participants came from cohorts established in eight regions within five European countries (Italy, UK, the Netherlands, Germany, Denmark) participating in the European Prospective Investigation into Cancer and Nutrition (EPIC) study [25].The cohorts were those in the EPIC that had a follow-up program including reassessment of anthropometry completed. Individuals were eligible if the following inclusion criteria were met: younger than 60 years of age at baseline and younger than 65 years at follow-up, blood sample available, had baseline information on diet, weight and height and follow-up information on weight, stable smoking habits, no cancer, cardiovascular diseases (CVD), and diabetes, and an annual weight change not more than 5 kg/year. A reported or recorded weight gain exceeding 5 kg/year is very unlikely to correspond to increased adiposity but rather much more likely to either being caused by an error in the measurement, in the data or emergence of a disease that induces water retention as oedema or ascites. As such, a total of 50,293 men and women out of 146,543 initially recruited participants were eligible to participate in our study.

Cases were defined as those individuals who had experienced the greatest degree of unexplained annual weight gain during follow-up (with an average duration of 6–8 years). They were identified by using the residuals from a regression model of annual weight change on baseline values of age, weight and height, smoking status (current/former/never smokers), and follow-up time. Regression models were run separately for each sex-country strata. For each of the five countries, except Italy, we selected 600 male and 600 female cases. As the Italian cohort consisted of a general population-based sample and of a women-only sample (population-based breast cancer screening program), men were underrepresented (27%). Approximately consistent with the sex-ratio in the Italian cohort, we selected 300 male and 900 female cases. In addition to this a random subcohort (RSC) sample was selected comprising a random sample of the total eligible cohort, drawn in such a way that the total number of noncases should generally equal the number of cases (with respect to number of individuals and sex-strata distribution). Since the original case-group sized stratified random samples resulted in some overlap of cases, in practice this was performed by random oversampling of noncases, except in Denmark where overlap between cases and subcohort was negligible (n  =  79). In total, 11,921 participants were included in the present genetic association study: 6,000 cases and a subcohort of 7,061 individuals, of which 5,921 were noncases. The demographic, anthropometric and dietary characteristics of cases, noncases and random subcohort are presented in Table 1. We used both a case-noncase group and a random subcohort group to be able to test for associations with different obesity related parameters–a categorical (dichotomous) variable in the case-control group and quantitative variables in the random sub cohort.

**Table 1:** Characteristics of participants of cases, noncases and subcohort.

	Cases (n = 5584)	Noncases (n = 5507)	*p-*values^1^	Subcohort (n = 6566)
Age, *yrs*	47.6±7.5	48.0±7.3	0.003	47.9±7.3
Sex, *%men*	45	45	matched	46
Overweight, *%*	43	39	<0.0001	39
Obesity, *%*	17	9	<0.0001	10
Baseline weight, *kg*	76.3±14.3	72.6±13.4	<0.0001	73.2±13.6
Baseline BMI, *kg/m^2^*	26.4±4.2	25.2±3.6	<0.0001	25.4±3.7
Annual weight change, *g/yr*	1,428±684	30±622	<0.0001	245±801
BMI at follow-up, *kg/m^2^*	29.4±4.4	25.3±3.5	<0.0001	25.9±3.9
Follow-up time, *yrs*	6.8±2.5	6.8±2.5	0.08	6.9±2.5
Glycemic index (GI)	56.6±4.3	56.5±4.1	0.4	56.5±4.1
Protein intake, *g*	89.9±29.4	89.2±27.1	0.2	89.6±28.2

Values presented are mean ± standard deviation or percentage (%) as indicated.

1 *p-*values for the difference between cases and noncases, tested by Student t-test (for continuous variables) or Cochran-Armitage trend test (categorical variables).

### Measurements of Diet, Anthropometrics and Smoking Status

Validated country-specific food frequency questionnaires (FFQs) were used to collect dietary information at baseline [25] on GI and protein intake, assessed using the methods described earlier [30], [31], and energy density.

Details of the anthropometric measurements have been described previously [30], [31]. In brief, at baseline all participants were measured for weight and height using standard study protocols [32]. At follow-up, participants in the UK and one center in the Netherlands (Doetinchem) were measured again by trained technicians, while all other participants measured their weight at home according to the guidance provided. Therefore participants from Doetinchem were analyzed separately from other Dutch participants. As such, we analyzed the data from six study centers in five countries. The cohort from Norfolk and one of the Dutch cohorts used objective measurement, but in the analyses of the various associations between baseline factors and weight gain, there were no indication that the association results were systematically different from the cohorts in which self-measured weight were used [27], [30], [31], [33].

Information on smoking status (never, former, or current smoker) was collected via self-administered questionnaires at baseline and at follow-up. Only those who had not changed their smoking habits during follow-up were included in the analyses.

### Selection of Candidate Genes and tagSNPs

We originally used the International HapMap data for European ancestry (CEU) (release 20, NCBI Build 35) to select SNPs such that full coverage of the common genetic variation in the *CTSS* gene (+/−5 kb) was ensured. We later checked that we still covered 100% of genetic variability with the latest HapMap version (HapMap Data Rel 27 Phase II + III, Feb 09 on NCBI B36 assembly, dbSNP B126).

The Haploview software V3.3 was used to assess the linkage disequilibrium (LD) structure between SNPs [34]. Tagger software was used to select tagSNPs with the ‘pairwise tagging only’ option and an LD measure r^2^ threshold of 0.8. In total, 4 SNPs were selected using the pre-requisite criteria based on the minor allele frequency (MAF) and Hardy-Weinberg Equilibrium (HWE): MAF≥5%, *p_HWE_* > 0.01.

### DNA Extraction and Genotyping

Genomic DNA was extracted from the buffy coats with a salting out method [35], except for participants from the UK, for whom whole-genome amplified DNA was used. Genomic and amplified DNA samples were quality-checked, quantified and normalized to approximately 100 ng/ml and 2.0 µg before genotyping. High throughput SNP genotyping was carried out using the Illumina® GoldenGate Genotyping System at IntegraGen, France.

We subjected all SNPs to country-specific HWE genotype distribution-tests. Significant deviations from equilibrium were defined as *p_HWE_* ≤ 0.001. This threshold was chosen in order to be concordant with other genetic studies carried out in the DiOGenes project. All four SNPs passed the tests for each country and were successfully genotyped for 11,091 participants. The case group included 5,584 participants and the random subcohort included 6,566 participants of whom 5,507 were noncases (Table 1).

### Genetic Variability at the *CTSS* Loci

**Figure 1:**
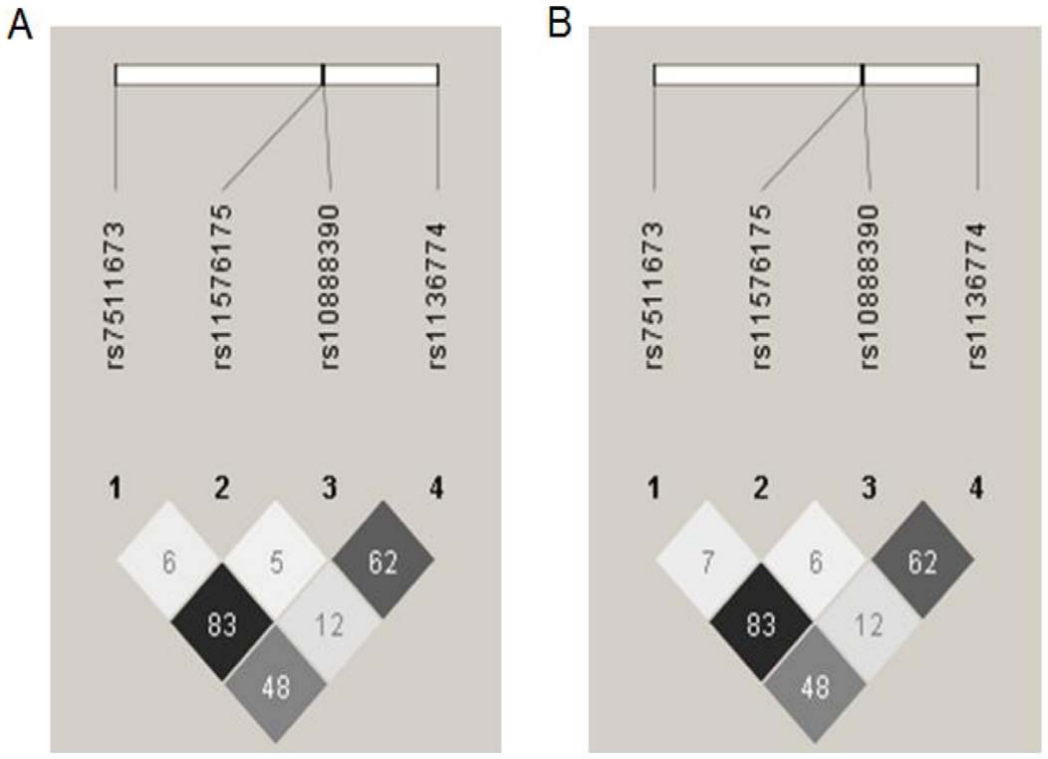
Linkage disequilibrium (LD) plot of the *CTSS* locus in cases and random subcohort. This Figure shows LD (linkage disequilibrium) values (r^2^) between each tag SNP in (A) cases and (B) subcohort. Each diamond contains the LD value (r^2^) between the two SNPs that face each of the upper sides of the diamond, ex: the LD between rs10888390 (SNP N°3) and rs1136774 (SNP N°4) is r^2^ = 0.62; the darker the diamond, the higher the LD value. There seems to be no difference in the LD pattern at the *CTSS* locus between the cases and the subcohort.

Four tag SNPs were selected in order to obtain a full coverage of the common variability at the *CTSS* locus +/−5 kb (chromosome 1, 1q21, position 148964178 to 149009929) in the HapMap CEU population. According to the latest HapMap Data Rel 27 Phase II + III, Feb 09 on NCBI B36 assembly dbSNP B126, rs7511673 (SNP N°1) captured 7 other SNPs–rs1415148, rs12089989, rs7418501, rs7521898, rs7540874, rs12086472 and rs11587444; rs11576175 (SNP N°2) captured no other SNP, rs10888390 (SNP N°3) captured 6 other SNPs–rs2275235, rs11204722, rs16827671, rs3768018, rs4537557 and rs10888391; and rs1136774 (SNP N°4) captured 2 other SNPs–rs12568757 and rs11204725. Figure 1 shows the LD pattern for the 4 selected tag SNPs in cases and RSC respectively. There seems to be no difference in the LD pattern at the *CTSS* locus between the cases and RSC. Two tag SNPs–rs7511373 and rs10888390 (SNP N°3)–are in strong LD in these two groups (r^2^ = 0.83 in both groups). Table 2 provides Hardy-Weinberg P-values, frequencies and counts for genotypes and alleles for the 4 SNPs investigated in this study both for the cases and the RSC. None of these SNPs significantly deviated from Hardy-Weinberg equilibrium in both the cases and the RSC (all p_HWE_>0.05).

**Table 2:** Description of *CTSS* variability in subcohort and cases.

*CTSS*							
		random subcohort			Cases		
		n	frequency	*p_HWE_*	n	frequency	*p_HWE_*
rs7511673	A/A	2382	0.36	0.93	2016	0.36	0.49
	A/T	3142	0.48		2699	0.48	
	T/T	1041	0.16		869	0.16	
	A	7906	0.60		6731	0.60	
	T	5224	0.40		4437	0.40	
rs11576175	G/G	5341	0.81	0.39	4571	0.82	0.84
	G/A	1155	0.18		960	0.17	
	A/A	70	0.01		52	0.01	
	G	11837	0.90		10102	0.90	
	A	1295	0.10		1064	0.10	
rs10888390	G/G	2721	0.41	0.69	2320	0.42	0.63
	G/A	2999	0.46		2544	0.46	
	A/A	844	0.13		717	0.13	
	G	8441	0.64		7184	0.64	
	A	4687	0.36		3978	0.36	
rs1136774	A/A	1833	0.28	0.76	1606	0.29	0.15
	A/G	3283	0.50		2727	0.49	
	G/G	1448	0.22		1250	0.22	
	A	6949	0.53		5939	0.53	
	G	6179	0.47		5227	0.47	

Genotype and allele counts, genotype and allele frequencies and Hardy Weinberg Equilibrium test p-values for each SNP in the subcohort and in the cases respectively.

### Statistical Methods

Each SNP was coded 0, 1 and 2 according to the number of minor alleles an individual carries (0 for those homozygous for the common allele, 1 for heterozygote and 2 for those homozygous for the minor allele).

First, the association between each SNP and each quantitative phenotype was tested using linear regression, assuming an additive effect of the minor allele. Second, we tested for SNP-dietary interaction associations with quantitative phenotype in the same manner. Third, case-noncase (CNC) logistic regression analyses were run, investigating possible SNP main effects on case-status (i.e. based on the risk of being a weight-gainer in the sense outlined above). These logistic regression analyses were then repeated as described above but including SNP-dietary effects.

CNC analyses of main effects were not adjusted, whereas RSC analyses were adjusted for variables that had been included in the case-status defining model (i.e. baseline values of age, height, sex, smoking status, and follow-up time) to reduce the residual variation and potential confounding. SNP-dietary variable interaction analyses were performed by including the corresponding interaction term as well as the complementary dietary main effect term in the model. Finally, change-based analyses were additionally adjusted for corresponding baseline values (additionally including baseline BMI when considering waist circumference for given BMI), and follow-up time was not used for adjustment when considering the cross-sectional (baseline) analyses.

All association analyses were first conducted for each study center separately and then effect-estimates were meta-analyzed. We used random effects to account for the possible heterogeneity across study centers, which presence was tested for using the Cochran Q-test [36].

Nominally significant associations (p<0.05) were retested assuming a dominant and a recessive model in the same way as described above.

All association analyses were conducted using Stata 9.2/11.1 for Windows (StataCorp LP, Texas, USA). The descriptive analyses were performed with SAS 9.1 for Windows (SAS Institute, Cary, NC).

Power calculations were performed using QUANTO software, Version 1.2.4 (May 2009) [37]. In the CNC analysis, the minimum detectable main effects, at 80% power, were ORs (odds ratios) 1.08 for rs7511673 (SNP N°1), 1.13 for rs11576175 (SNP N°2), and 1.08 for both rs10888390 (SNP N°3) and rs1136774 (SNP N°4). In the RSC analysis, the minimum detectable main effects, at 80% power, for weight change during the study, were regression coefficients (β) 40 g/y for rs7511673 (SNP N°1), 66 g/y for rs11576175 (SNP N°2), 41 g/y for rs10888390 (SNP N°3) and 40 g/y for rs1136774 (SNP N°4).

## Results

### Association between *CTSS* SNPs and BMI at Baseline

We found that the minor allele of rs11576175 (SNP N°2) was associated with lower BMI at baseline (p = 0.02, β = −0.24, Figure S1, Table 3). When tested assuming a dominant model, the association was also significant (p = 0.01, β = −0.29, Table S1).

**Table 3:** Associations between *CTSS* SNPs, BMI and body fat distribution at baseline.

SNP	Phenotype	Estimate	*P*	SE	CI 95% lower	CI 95% higher
rs7511673 (SNP N°1)	BMI	−0.03	0.62	0.06	−0.15	0.09
	Waist	−0.08	0.65	0.16	−0.40	0.25
	Waist for given BMI	0.00	0.95	0.08	−0.16	0.15
rs11576175 (SNP N°2)	BMI	−0.24	**0.02**	0.10	−0.45	−0.04
	Waist	−0.50	0.06	0.27	−1.03	0.02
	Waist for given BMI	0.13	0.31	0.13	−0.13	0.39
rs10888390 (SNP N°3)	BMI	−0.01	0.93	0.07	−0.14	0.13
	Waist	0.00	1.00	0.17	−0.33	0.33
	Waist for given BMI	0.03	0.67	0.08	−0.13	0.20
rs1136774 (SNP N°4)	BMI	−0.09	0.22	0.07	−0.23	0.05
	Waist	−0.16	0.36	0.18	−0.51	0.19
	Waist for given BMI	0.08	0.30	0.08	−0.07	0.24

Overall Meta analysis estimates (β), p values, standard error and 95% confidence intervals for association between SNPs and BMI and body fat distribution at baseline in the random subcohort.

### Association between *CTSS* SNPs and Body Fat Distribution at Baseline

No significant association between studied SNPs and body fat distribution were found (Table 3).

### Association between *CTSS* SNPs and Annual Weight Change

The interaction between rs11576175 (SNP N°2) and the percentage of proteins contained in the diet was significantly associated to case-status (interaction p = 0.01, OR = 1.05, Table 4). For each additional minor allele, the estimated risk of being a weight gainer increases by 1.05 odds per extra one percent of proteins in the diet. This association was also significant in this population when assuming a dominant model (p = 0.004, OR = 1.06, Table S1).

**Table 4:** Association between *CTSS* SNPs and weight change during the study.

SNP	Phenotype	Effect	Estimate	*P*	SE	CI 95% lower	CI 95% higher
rs7511673 (SNP N°1)	Weight (RSC)	main effect	−15.52	0.19	11.85	−38.74	7.70
		interaction diet ED	−31.53	0.47	44.12	−117.99	54.94
		interaction diet GI	−2.79	0.42	3.49	−9.63	4.05
		interaction diet protein	−0.22	0.61	0.44	−1.08	0.64
		interaction diet protein %	−3.48	0.49	5.09	−13.46	6.51
	Case/noncase	main effect	0.99	0.79	0.03	0.93	1.06
		interaction diet ED	0.94	0.50	0.10	0.77	1.13
		interaction diet GI	0.99	0.17	0.01	0.97	1.00
		interaction diet protein	1.00	0.47	0.00	1.00	1.00
		interaction diet protein %	1.00	0.98	0.01	0.97	1.03
rs11576175 (SNP N°2)	Weight (RSC)	main effect	31.71	0.09	18.89	−5.32	68.73
		interaction diet ED	−13.88	0.85	73.59	−158.11	130.36
		interaction diet GI	−1.62	0.81	6.63	−14.62	11.37
		interaction diet protein	0.18	0.87	1.06	−1.89	2.25
		interaction diet protein %	5.60	0.53	8.93	−11.91	23.10
	Case/noncase	main effect	0.99	0.75	0.05	0.90	1.08
		interaction diet ED	0.94	0.77	0.20	0.63	1.40
		interaction diet GI	1.00	0.99	0.02	0.97	1.03
		interaction diet protein	1.00	0.14	0.00	1.00	1.01
		interaction diet protein %	1.05	**0.01**	0.02	1.01	1.09
rs10888390 (SNP N°3)	Weight (RSC)	main effect	−10.13	0.40	12.09	−33.83	13.57
		interaction diet ED	−20.92	0.64	44.92	−108.96	67.13
		interaction diet GI	0.13	0.98	4.42	−8.54	8.79
		interaction diet protein	−0.24	0.66	0.53	−1.28	0.81
		interaction diet protein %	−2.73	0.64	5.89	−14.26	8.81
	Case/noncase	main effect	0.99	0.85	0.04	0.92	1.07
		interaction diet ED	0.91	0.34	0.10	0.75	1.10
		interaction diet GI	0.99	0.24	0.01	0.97	1.01
		interaction diet protein	1.00	0.23	0.00	1.00	1.00
		interaction diet protein %	1.00	0.76	0.01	0.98	1.03
rs1136774 (SNP N°4)	Weight (RSC)	main effect	5.51	0.64	11.64	−17.30	28.32
		interaction diet ED	−40.39	0.35	43.40	−125.45	44.67
		interaction diet GI	−1.03	0.82	4.46	−9.78	7.72
		interaction diet protein	−0.02	0.98	0.85	−1.69	1.65
		interaction diet protein %	0.25	0.97	6.75	−12.98	13.48
	Case/noncase	main effect	1.00	0.90	0.03	0.95	1.05
		interaction diet ED	0.90	0.28	0.09	0.75	1.09
		interaction diet GI	0.99	0.23	0.01	0.98	1.01
		interaction diet protein	1.00	0.96	0.00	1.00	1.00
		interaction diet protein %	1.02	0.23	0.01	0.99	1.04

Overall Meta analysis estimates (β or odd ratios), p values, standard error and 95% confidence intervals for association between SNPs and weight change during the study, ED: energy density, GI: glycemic index. RSC: random subcohort.

### Association between *CTSS* SNPs and Annual Body Fat Distribution Change

Both rs7511673 (SNP N°1) and rs10888390 (SNP N°3) were associated with annual waist change (p = 0.01, β = −0.04, Figure S2, Table 5 and p = 0.04, β = −0.03, Figure S3, Table 5 respectively). Rs7511673 (SNP N°1) was associated with a change in waist circumference of 0.04 cm per year and per minor allele and rs10888390 (SNP N°3) was associated with a change in waist circumference of 0.03 cm per year and per minor allele. Nevertheless these two SNPs are in strong LD in our populations (r^2^ = 0.83, Figure 1). The association between rs7511673 (SNP N°1) and waist gain was significant when assuming a dominant model (p =  0.02, β = −0.06, Table S1). Rs7511673 (SNP N°1) was also associated with change in waist circumference for given BMI (p = 0.03, β = −0.03, Figure S4, Table 5)–rs7511673 (SNP N°1) was associated with a change in waist circumference of 0.03 cm per year and per minor allele. This association was significant when assuming a dominant model (p = 0.02, β = −0.04, Table S1).

**Table 5:** Association between *CTSS* SNPs and body fat distribution change during the study.

SNP	Phenotype	Effect	Estimate	*P*	SE	CI 95% lower	CI 95% higher
rs7511673 (SNP N°1)	Waist (RSC)	main effect	−0.04	**0.01**	0.02	−0.08	−0.01
		interaction diet ED	0.02	0.75	0.06	−0.10	0.14
		interaction diet GI	0.00	0.75	0.01	−0.02	0.01
		interaction diet protein	0.00	0.43	0.00	0.00	0.00
		interaction diet protein %	0.00	0.79	0.01	−0.02	0.01
	Waist for given BMI	main effect	−0.03	**0.03**	0.01	−0.05	0.00
	(RSC)	interaction diet ED	0.04	0.44	0.05	−0.05	0.12
		interaction diet GI	0.00	0.72	0.01	−0.02	0.01
		interaction diet protein	0.00	0.26	0.00	0.00	0.00
		interaction diet protein %	0.00	0.61	0.01	−0.01	0.01
rs11576175 (SNP N°2)	Waist (RSC)	main effect	0.01	0.82	0.03	−0.05	0.06
		interaction diet ED	−0.01	0.91	0.10	−0.21	0.19
		interaction diet GI	0.00	0.68	0.01	−0.03	0.02
		interaction diet protein	0.00	0.31	0.00	0.00	0.00
		interaction diet protein %	0.02	0.16	0.01	−0.01	0.04
	Waist for given BMI	main effect	0.00	0.98	0.02	−0.04	0.04
	(RSC)	interaction diet ED	−0.05	0.50	0.07	−0.20	0.10
		interaction diet GI	−0.01	0.24	0.01	−0.02	0.01
		interaction diet protein	0.00	0.32	0.00	0.00	0.00
		interaction diet protein %	0.02	0.14	0.01	−0.01	0.04
rs10888390 (SNP N°3)	Waist (RSC)	main effect	−0.03	**0.04**	0.02	−0.07	0.00
		interaction diet ED	0.02	0.72	0.06	−0.10	0.15
		interaction diet GI	0.00	0.95	0.01	−0.01	0.02
		interaction diet protein	0.00	0.31	0.00	0.00	0.00
		interaction diet protein %	0.00	0.85	0.01	−0.01	0.02
	Waist for given BMI	main effect	−0.02	0.06	0.01	−0.05	0.00
	(RSC)	interaction diet ED	0.03	0.48	0.05	−0.06	0.12
		interaction diet GI	0.00	0.71	0.01	−0.02	0.01
		interaction diet protein	0.00	0.22	0.00	0.00	0.00
		interaction diet protein %	0.00	0.90	0.01	−0.01	0.01
rs1136774 (SNP N°4)	Waist (RSC)	main effect	−0.02	0.18	0.02	−0.05	0.01
		interaction diet ED	−0.01	0.92	0.06	−0.13	0.11
		interaction diet GI	0.00	0.73	0.01	−0.01	0.01
		interaction diet protein	0.00	0.96	0.00	0.00	0.00
		interaction diet protein %	0.01	0.43	0.01	−0.01	0.02
	Waist for given BMI	main effect	−0.02	0.24	0.01	−0.04	0.01
	(RSC)	interaction diet ED	0.00	0.96	0.05	−0.10	0.09
		interaction diet GI	0.00	0.53	0.01	−0.02	0.01
		interaction diet protein	0.00	0.36	0.00	0.00	0.00
		interaction diet protein %	0.00	0.70	0.01	−0.01	0.01

Overall Meta analysis estimates (β or odd ratios), p values, standard error and 95% confidence intervals for associations between SNPs and body fat distribution change during the study, ED: energy density, GI: glycemic index. RSC: random subcohort.

## Discussion

In this study we found several associations between *CTSS* polymorphisms and anthropometric parameters including baseline BMI (rs11576175 (SNP N°2)), waist change over time (rs7511673 (SNP N°1) and rs10888390 (SNP N°3)). Although this waist change (0.03–0.04 cm/yr) is unlikely to have clinical relevance if considered on its own, this association should rather be considered in combination with other risk factors. Importantly rs11576175 (SNP N°2) was also associated with the risk of being a weight gainer, and this association was under the influence of the percentage of proteins contained in the diet. Rs7511673 (SNP N°1) captured 7 other SNPs and rs10888390 (SNP N°3) captured 6 other SNPs, besides this, two tag SNPs–rs7511673 (SNP N°1) and rs10888390 (SNP N°3)–are in LD in both of our study groups (r^2^ = 0.83 in each group), which means that any association with one of these variants could be caused by one of at least 14 other SNPs. There is a controversy regarding the role of fat intake on obesity related phenotypes–some studies found that fat intake had an important role [38] whereas others found that it had no importance at all [39]–[49]. Furthermore a study carried out in the EPIC cohorts, which investigated the role of fat intake on body weight change yielded no significant association between the type or amount of dietary fat and weight change [50]. For this reason, we decided not to investigate the interaction between *CTSS* SNPs and the type or amount of dietary fat in our study.

Many statistical tests have been performed therefore the question of multiple testing should be raised. The p-values presented in our study are uncorrected in order to avoid conservative corrections and loss of power (after correcting by an FDR adjustment [51] (data not shown) none of the p-values were significant). A further – although largely overlapping – motivation for not restricting the presentation and discussion to p-values adjusted for multiple comparisons is that our study is exploratory; therefore our results will need to be replicated in large independent cohorts (for related discussion, see e.g. [52], [53]).

Our group has previously published an association between *CTSS* variants and lipid metabolism related parameters [24]. In addition, we identified an association between a genetic variant located in *CST3*, a gene coding for an endogenous inhibitor of Cathepsin S, and BMI measured repeatedly during lifetime in independent European populations [18]. These observations suggest that potential alterations of Cathepsin pathway, eventually genetically induced, might contribute to changes in corpulence over time and are therefore consistent with the observations reported in this present paper. The obesity related phenotypes of *CTSK*−/− [15] and *CTSL*−/− [16] mice are also in agreement with this hypothesis [17]. Fontanesi et al [54] found an association between a *CTSS* polymorphism and feed:gain ratio and average daily gain in a group of Italian large white pigs. These findings seem to be in agreement with ours.

Noteworthy, *CTSS* has not been identified as associated to obesity related parameters by the large GWAS [2], [9]. However this may be due to the fact that these studies focus on one time point and do not investigate longitudinal data, therefore the genes that influence changes in corpulence may not be detectable by these approaches. Moreover, these studies do not account for dietary habits. Finally, it might be that these associations were not identified by GWAS simply because of the small effect size of the associations–although GWAS include many more individuals than in our study, the significance level that is generally applied in GWAS is much lower than the one applied in our study (0.05). We cannot exclude that these associations are caused by one or several variants acting on a gene nearby *CTSS*. *CTSK*, the gene that codes for Cathepsin K, an enzyme that is also involved in obesity [17], is located in the same genomic region as *CTSS* (1q21) [55]–[57]. In the HapMap CEU population, *CTSS*rs11576175 (SNP N°2) is in perfect LD with *CTSK*rs4379678 (r^2^ = 1), which means that the associations we found with rs11576175 (SNP N°2) might actually reflect an association with rs4379678. Furthermore we have identified a complex association between rs11576175 (SNP N°2) and the risk of being a weight gainer–the interaction between rs11576175 (SNP N°2) and the percentage of proteins in the diet was associated with the risk of being a weight gainer. A potential link between high protein diet and improved weight and fat loss has been reported [58]. These observations may be explained by the fact that proteins might be more satiating than fat or carbohydrate [59]. Very little is known concerning the molecular mechanisms underlying this process and especially regarding the potential link between Cathepsins, and in particular Cathepsin S, and dietary protein intake. The possibility that dietary changes could influence the expression of Cathepsins has been highlighted by the outcomes of both animal models and *in vitro* studies. In mice, after infection by *Paracoccidioides brasiliensis* (a fungus that causes Paracoccidioidomycosis, a systemic mycosis), a very high protein diet was associated with a greater increase in spleen and liver Cathepsin G mRNA than a low protein diet [60]. Furthermore, *in vitro*, pyridoxal phosphate, a coenzyme form of vitamin B_6_, strongly inhibits Cathepsin B activity and weakly inhibits Cathepsin S and K activities [61].

In conclusion, we have identified nominally significant associations between several *CTSS* variants and obesity related parameters. One of these associations seems to be influenced by dietary protein intake. However this link needs to be further investigated in order to gain knowledge on the mechanisms governing weight homeostasis.

## Supporting Information

Figure S1 **BMI at baseline according to rs11576175 (SNP N°2).** Mean +/− SEM of BMI at baseline according to rs11576175 genotypes (G/G n = 5341, G/A n = 1155, and A/A n = 70) in the subcohort, n = 6566. Rs11576175 was associated with a decrease of 0.24 kg/m^2^ per A allele (p = 0.02, β = −0.24).
(TIF)

Figure S2 **Annual waist gain according to rs7511673 (SNP N°1).** Mean +/- SEM of annual waist gain according to rs7511673 genotypes (A/A n = 2382, A/T, n = 3142, and T/T, n = 1041) in the subcohort, n = 6566. In the regression analysis rs7511673 was associated with a decrease in waist circumference of 0.04 cm per year and per T allele (p = 0.01, β = −0.04). This association was also significant when assuming a dominant model (p = 0.02, β = −0.06), A/T and T/T carriers gained 0.06 cm per year less than A/A carriers.
(TIF)

Figure S3 **Annual waist gain according to rs10888390 (SNP N°3).** Mean +/− SEM of annual waist gain according to rs10888390 genotypes (G/G n = 2721, G/A n = 2999, and A/A n = 844) in the subcohort, n = 6566. rs10888390 was associated with a decrease in waist circumference of 0.03 cm per year and per A allele (p = 0.04, β = −0.03).
(TIF)

Figure S4 **Annual waist for given BMI gain per year according to rs7511673 (SNP N°1).** Mean +/− SEM of annual waist gain for given BMI according to rs7511673 genotypes (A/A n = 2382, A/T, n = 3142, and T/T, n = 1041) in the subcohort, n = 6566. Rs7511673 was associated with a decrease in waist circumference for given BMI of 0.03 cm per year and per T allele (p = 0.03, β = −0.03). This association was also significant when assuming a dominant model (p = 0.02, β = −0.04).
(TIF)

Table S1 **Dominant and recessive models for associations which were significant when assuming an additive model.**
(DOC)
