# Supplementary figures and images for: Dietary Factors Impact on the Association between CTSS Variants and Obesity Related Traits
Source: PLoS One. 2012 Jul 23;7(7):e40394. doi: 10.1371/journal.pone.0040394 (PMC3402491; doi:10.1371/journal.pone.0040394)

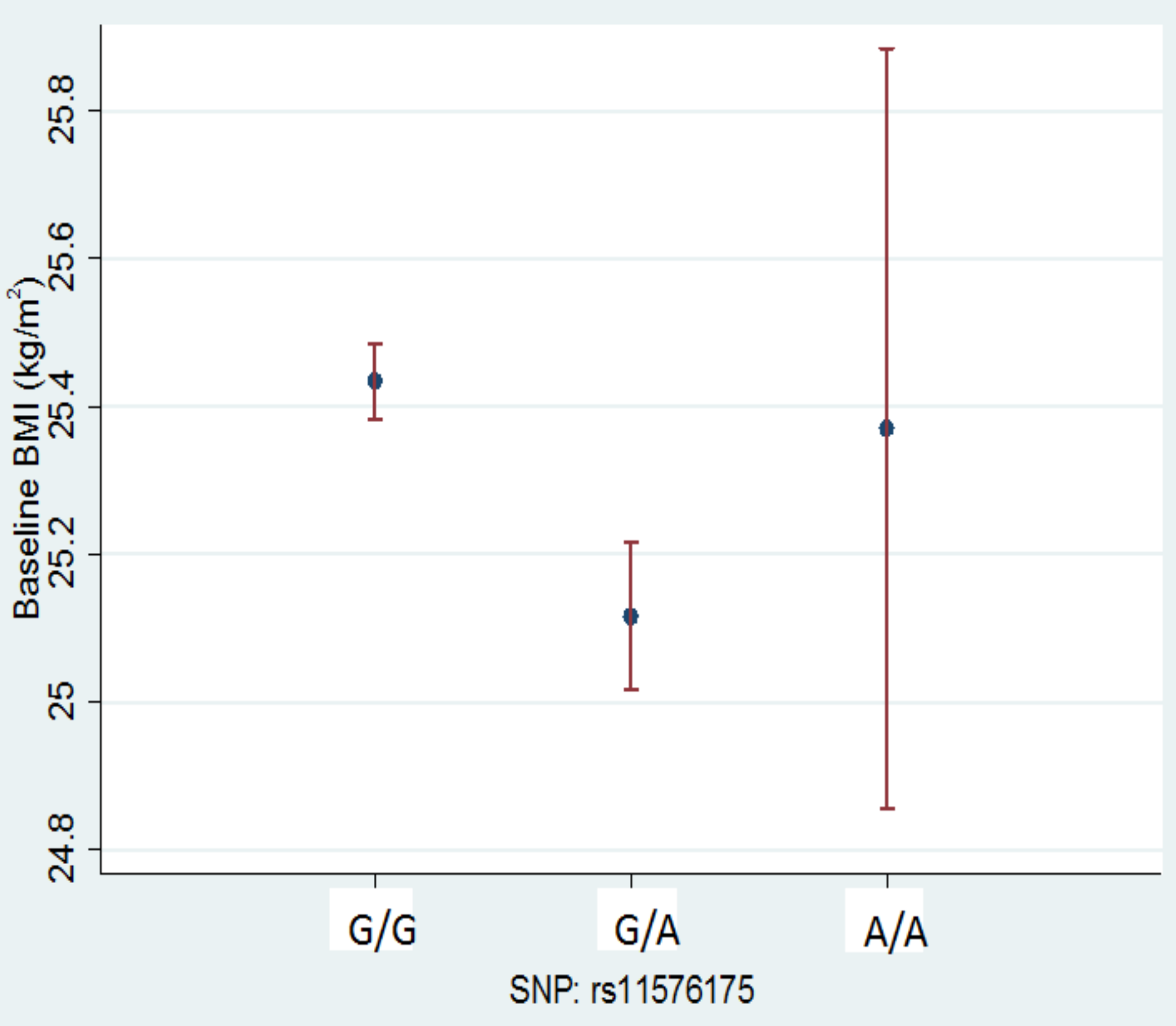

Supplement: Figure S1 — BMI at baseline according to rs11576175 (SNP N°2). Mean +/− SEM of BMI at baseline according to rs11576175 genotypes (G/G n = 5341, G/A n = 1155, and A/A n = 70) in the subcohort, n = 6566. Rs11576175 was associated with a decrease of 0.24 kg/m2 per A allele (p = 0.02, β = −0.24). (TIF) [file pone.0040394.s001.tif]

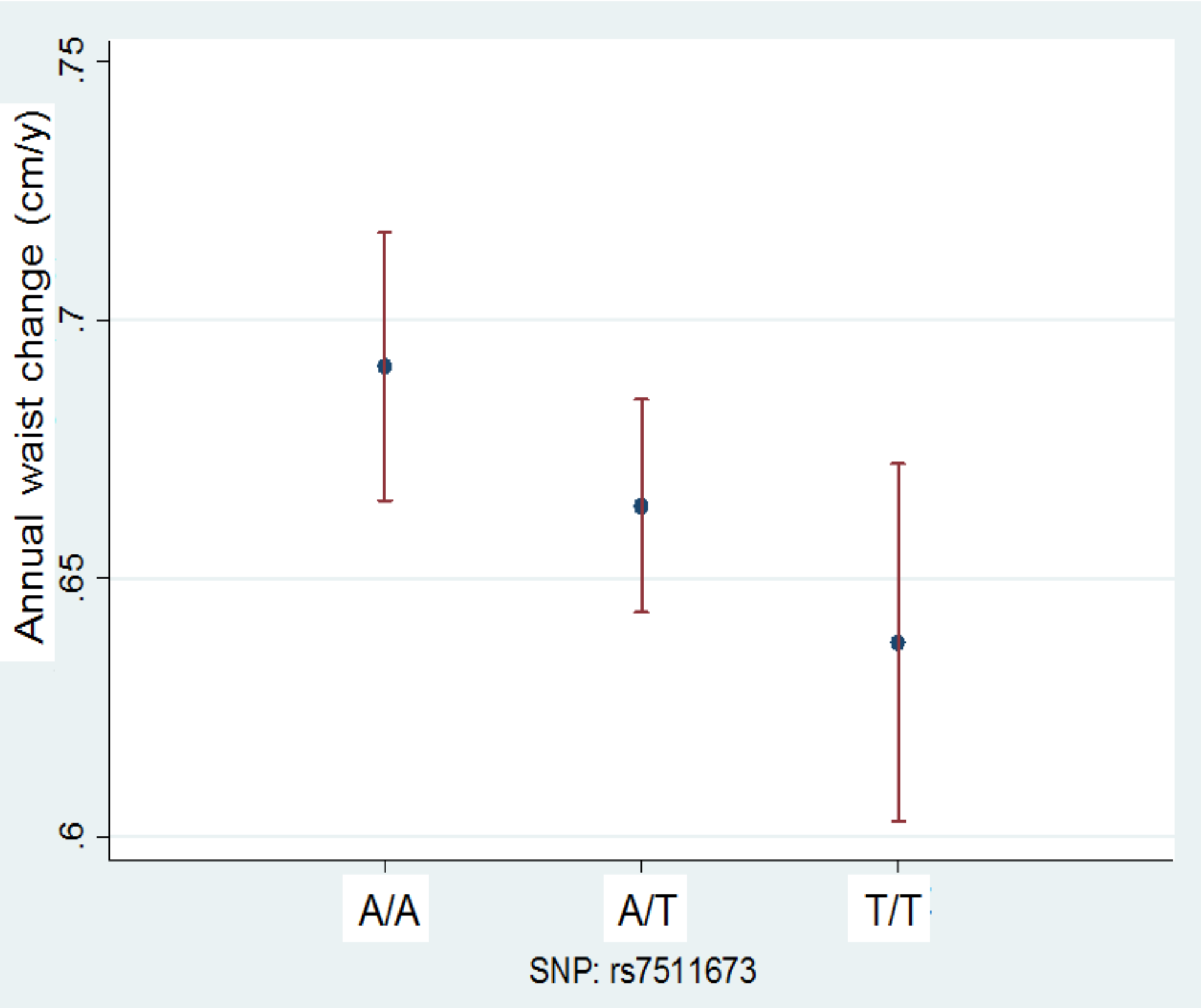

Supplement: Figure S2 — Annual waist gain according to rs7511673 (SNP N°1). Mean +/- SEM of annual waist gain according to rs7511673 genotypes (A/A n = 2382, A/T, n = 3142, and T/T, n = 1041) in the subcohort, n = 6566. In the regression analysis rs7511673 was associated with a decrease in waist circumference of 0.04 cm per year and per T allele (p = 0.01, β = −0.04). This association was also significant when assuming a dominant model (p = 0.02, β = −0.06), A/T and T/T carriers gained 0.06 cm per year less than A/A carriers. (TIF) [file pone.0040394.s002.tif]

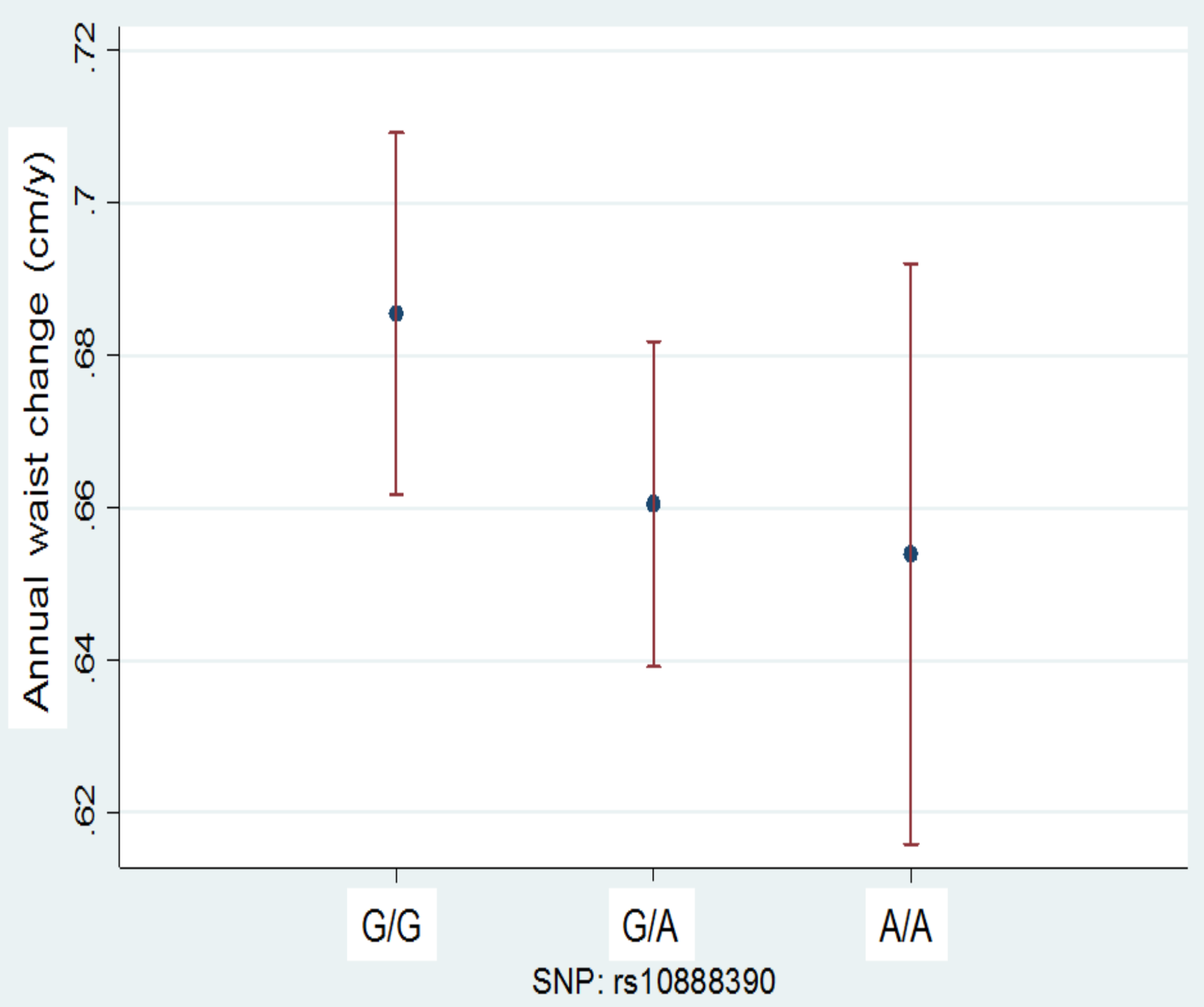

Supplement: Figure S3 — Annual waist gain according to rs10888390 (SNP N°3). Mean +/− SEM of annual waist gain according to rs10888390 genotypes (G/G n = 2721, G/A n = 2999, and A/A n = 844) in the subcohort, n = 6566. rs10888390 was associated with a decrease in waist circumference of 0.03 cm per year and per A allele (p = 0.04, β = −0.03). (TIF) [file pone.0040394.s003.tif]

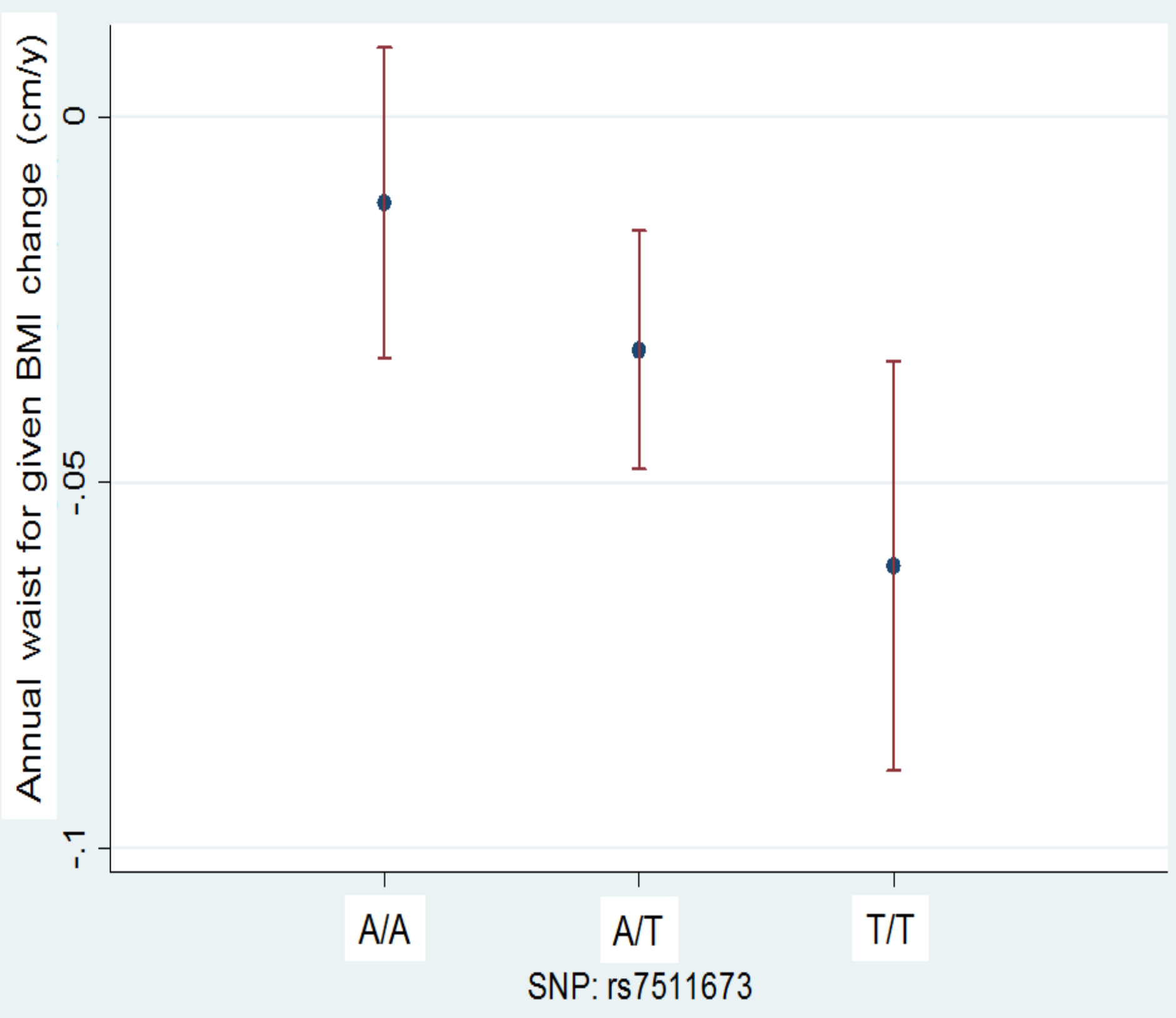

Supplement: Figure S4 — Annual waist for given BMI gain per year according to rs7511673 (SNP N°1). Mean +/− SEM of annual waist gain for given BMI according to rs7511673 genotypes (A/A n = 2382, A/T, n = 3142, and T/T, n = 1041) in the subcohort, n = 6566. Rs7511673 was associated with a decrease in waist circumference for given BMI of 0.03 cm per year and per T allele (p = 0.03, β = −0.03). This association was also significant when assuming a dominant model (p = 0.02, β = −0.04). (TIF) [file pone.0040394.s004.tif]
