# Supplementary material for: Dietary Factors Impact on the Association between CTSS Variants and Obesity Related Traits
Source: PLoS One. 2012 Jul 23;7(7):e40394. doi: 10.1371/journal.pone.0040394 (PMC3402491; doi:10.1371/journal.pone.0040394)
Supplement: Table S1 — Dominant and recessive models for associations which were significant when assuming an additive model. (DOC) [file pone.0040394.s005.doc]

**Table S1. Dominant and recessive models for associations which were significant when assuming an additive model.**

| **SNP** | **Phenotype** | **Effect** | **Model** | **Estimate** | *p* | **SE** | **CI 95% lower** | **CI 95% higher** |
| --- | --- | --- | --- | --- | --- | --- | --- | --- |
| rs7511673 (SNP N°1) | Waist gain | Main effect | Dominant | -0.0562 | **0.02** | 0.0241 | -0.1034 | -0.0090 |
| rs7511673 (SNP N°1) | Waist gain | Main effect | Recessive | -0.0578 | 0.07 | 0.0315 | -0.1196 | 0.0040 |
| rs7511673 (SNP N°1) | Waist for given BMI gain | Main effect | Dominant | -0.0404 | **0.02** | 0.0176 | -0.0749 | -0.0059 |
| rs7511673 (SNP N°1) | Waist for given BMI gain | Main effect | Recessive | -0.0268 | 0.24 | 0.0230 | -0.0719 | 0.0183 |
| rs11576175 (SNP N°2) | Weight baseline | Main effect | Dominant | -0.8234 | **0.01** | 0.3148 | -1.4403 | -0.2064 |
| rs11576175 (SNP N°2) | Weight baseline | Main effect | Recessive | 0.0737 | 0.95 | 1.1789 | -2.2370 | 2.3844 |
| rs11576175 (SNP N°2) | Case/noncase | Interaction SNP diet protein content (%) | Dominant | 1.0621 | **<0.01** | 0.0211 | 1.0191 | 1.1070 |
| rs11576175 (SNP N°2) | Case/noncase | Interaction SNP diet protein content (%) | Recessive | 0.9376 | 0.52 | 0.1002 | 0.7704 | 1.1410 |
| rs10888390 (SNP N°3) | Waist gain | Main effect | Dominant | -0.0429 | 0.07 | 0.0235 | -0.0890 | 0.0032 |
| rs10888390 (SNP N°3) | Waist gain | Main effect | Recessive | -0.0475 | 0.17 | 0.0344 | -0.1149 | 0.0200 |

Overall Meta analysis estimates (β or odd ratios), p values, standard error and 95% confidence intervals assuming a dominant and recessive model for associations that were significant when assuming an additive model.
